# Gender differences in the relationship between peer victimization and non-suicidal self-injury in adolescents with depressive disorders

**DOI:** 10.3389/fpsyg.2025.1596144

**Published:** 2025-09-03

**Authors:** Aihua Yin, Shumiao Liu, Chunyan Wang, Yang Wang, Dongdong Qiao, Yongjie Zhou

**Affiliations:** ^1^Department of Psychiatry, Shandong Mental Health Center, Shandong University, Jinan, China; ^2^Shenzhen Mental Health Center, Shenzhen Kangning Hospital, Shenzhen, China

**Keywords:** non-suicidal self-injury, peer victimization, gender differences, depressive disorders, adolescence

## Abstract

**Background:**

Non-suicidal self-injury (NSSI) is common among adolescents with depressive disorders, yet the role of peer victimization and gender differences remains understudied. This study examines how different types of peer victimization (physical, verbal, social, property) relate to NSSI, focusing on gender as a moderator.

**Methods:**

A cross-sectional study of 2,343 adolescents with depressive disorders (517 males, 1,826 females) was conducted across 14 medical institutions in China. Peer victimization and NSSI were measured using the PVQ and C-FASM, respectively. Gender-stratified multiple linear regressions examined predictors of NSSI and its functions.

**Results:**

NSSI was more prevalent in females (80.18%) than males (61.51%) (*χ*^2^ = 77.094, *p* < 0.001), with higher scores for emotion regulation (*β* = 12.33 vs. 9.85, *p* < 0.001). Regression analyses revealed gender-specific predictors: in males, social victimization (*β* = 1.213, *p* = 0.036) and verbal victimization (*β* = 1.764, *p* = 0.031) significantly predicted NSSI related to emotion regulation; in females, physical victimization (*β* = 0.768, *p* = 0.005) and social victimization (*β* = 0.873, *p* < 0.001) were the primary predictors. For attention-seeking functions, physical, social, and verbal victimization were significant predictors in both genders, with higher coefficients observed in males. It is noteworthy that property victimization was significantly associated with social avoidance-driven NSSI only in females (*β* = 0.564, *p* = 0.001), highlighting the gender-specific impact of victimization on NSSI functions.

**Conclusion:**

Peer victimization is a significant predictor of NSSI, with clear gender-specific patterns. Gender-sensitive assessments and interventions tailored to victimization type are essential for early prevention.

## Introduction

Non-suicidal self-injury (NSSI) refers to deliberate self-inflicted damage to bodily tissues without suicidal intent (Nock, 2009). In recent years, the high prevalence of NSSI among adolescents has garnered significant attention, particularly within populations diagnosed with depressive disorders, where incidence rates are markedly elevated compared to their non-clinical peers (Muehlenkamp et al., 2012). Research has indicated that NSSI serves multiple functions in adolescents, including emotion regulation, attention-seeking, and social avoidance (Taylor et al., 2018). Previous studies have shown that external factors, such as dysfunctional family dynamics and insufficient social support, are closely related to NSSI in adolescents (Wang et al., 2022). However, the role of external social factors in the occurrence of NSSI remains insufficiently explored, particularly the impact of peer victimization.

Peer victimization refers to an individual experiencing physical, verbal, social, or property-related aggression or exclusion within peer relationships (Singham et al., 2017). Studies have demonstrated that peer victimization not only contributes to emotional distress, anxiety, and depression but may also serve as a significant risk factor for NSSI (van Geel et al., 2022). A meta-analysis by Huang et al. (2022) confirmed that peer victimization is a strong predictor of NSSI, highlighting that social victimization has a stronger impact on females, while physical victimization is more influential for males. This finding underscores the importance of considering gender differences in the victimization-NSSI link. Victimized individuals may engage in self-injury as a means of alleviating emotional distress, coping with social rejection, or eliciting attention from others (Klonsky, 2007). Different forms of victimization may influence NSSI through distinct mechanisms. For instance, social victimization (exclusion or ostracization by peers) may be associated with NSSI driven by social avoidance, whereas verbal victimization (experiencing verbal abuse) may exacerbate negative self-perception, leading to emotion-regulating NSSI (Platt et al., 2013).

Furthermore, gender may moderate the relationship between peer victimization and NSSI. Prior research has consistently shown that adolescent females exhibit a higher prevalence of NSSI than males, potentially due to their greater propensity to internalize emotional distress (Bresin and Schoenleber, 2015; Moloney et al., 2024). Additionally, cultural factors may shape how adolescents respond to peer victimization. You and Leung (2012) found that in Chinese adolescents, females are particularly sensitive to social exclusion, while males react more strongly to verbal insults. This cultural perspective aligns with the socialization patterns observed in Chinese society, where interpersonal relationships are highly valued, especially for females. Females are more likely to engage in NSSI as an emotion regulation strategy, whereas males tend to display externalizing behaviors such as aggression and impulsivity (Giletta et al., 2012). Additionally, gender differences exist in coping strategies when confronted with peer victimization. Females may be more sensitive to social and physical victimization, given the emphasis on interpersonal relationships in their socialization processes (Whitlock et al., 2013). Conversely, males may be more affected by verbal and social victimization, as societal norms often discourage emotional expression and help-seeking behaviors among them (van Geel et al., 2022).

Despite growing research on the relationship between peer victimization and NSSI, several gaps remain. First, most existing studies focus on general adolescent populations, with limited attention to adolescents diagnosed with depressive disorders. Given the well-established association between depressive disorders and NSSI (Xu et al., 2024), examining the role of peer victimization in this specific population is crucial for understanding NSSI’s underlying mechanisms. Second, the moderating effect of gender in the victimization-NSSI relationship remains underexplored. While previous studies suggest higher NSSI prevalence among females, the gender-specific responses to different forms of victimization require further investigation. Researches suggest that emotion dysregulation mediates the relationship between peer victimization and NSSI, with full mediation observed in females and partial mediation in males (Gu et al., 2025; Zhao et al., 2022; Liang et al., 2025). This indicates that the pathways linking victimization and NSSI may differ by gender, reinforcing the need to examine gender-specific mechanisms in this association. Lastly, most studies categorize NSSI as a binary variable (presence vs. absence), overlooking its functional heterogeneity—individuals may engage in NSSI for distinct psychological needs, such as emotion regulation, attention-seeking, or social avoidance (Taylor et al., 2018).

Therefore, this study aims to examine the relationship between various forms of peer victimization (physical, social, verbal, and property victimization) and NSSI, with a particular focus on gender as a moderating factor. Utilizing data from adolescents with depressive disorders across 14 medical institutions in nine provinces in China, this study employs standardized measures to assess victimization experiences and NSSI behaviors. Regression analysis will be conducted to explore the moderating role of gender in these associations. The findings of this study will contribute to a deeper understanding of the mechanisms underlying NSSI in adolescents with depressive disorders and provide empirical support for gender-specific intervention strategies. By clarifying the differential impacts of peer victimization on NSSI functions, this research aims to enhance the identification of high-risk individuals and inform the development of more targeted prevention strategies to reduce NSSI prevalence among adolescents.

## Methods

### Study sample

This study is a cross-sectional study designed to investigate the relationship between peer victimization and NSSI, with a particular focus on gender differences. A total of 2,343 adolescents with depressive disorders participated in this study, including 517 males (22.1%) and 1,826 females (77.9%). Participants were recruited from 14 general hospitals and mental health institutions across nine provinces in China. All participants were diagnosed with depressive disorders, including major depressive disorder (MDD), bipolar disorder-depressive episode (BD-D), or depressive state, by senior psychiatrists according to DSM-5 criteria.

To ensure sufficient statistical power, we conducted a sample size calculation based on an expected effect size (medium effect, Cohen’s *d* ≈ 0.5) and a conventional statistical power of 0.8. Using G*Power software, assuming an effect size of 0.5, a significance level of 0.05, and a power of 0.8, the required sample size was calculated to be approximately 200–300 participants. In the actual recruitment process, we recruited a total of 2,343 participants, including 517 males and 1,826 females.

The study was approved by the Ethics Committee of Shenzhen Kangning Hospital (IRB: 2020-k021-02) and the Ethics Committee of Shandong Mental Health Center (Approval No.: (2021) Lunshen R86).

Inclusion criteria were as follows: (i) Aged 12–18 years; (ii) Diagnosed with a depressive disorder (MDD, BD-D, or depressive state) according to DSM-5 criteria by senior psychiatrists; (iii) Adequate cognitive ability to complete the research questionnaire; (iv) Written informed consent was obtained from both the participant and their legal guardian. Exclusion criteria included: (i) Diagnosis of pervasive developmental disorders or intellectual disability; (ii) Presence of suicidal or homicidal ideation requiring immediate intervention; (iii) Active psychotic symptoms, such as schizophrenia or a manic episode; (iv) Receipt of systematic NSSI-related psychological therapy or pharmacological intervention within the past 6 months.

### Peer victimization

Peer victimization was assessed using the Chinese version of the Multidimensional Peer Victimization Questionnaire (PVQ) (Betts et al., 2015), which evaluates peer victimization experiences over the past semester. The PVQ consists of 16 items, rated on a 5-point Likert scale (0–4), with higher scores indicating greater severity of victimization. The scale includes four subdimensions, including physical victimization, social victimization, verbal victimization, and property victimization (Singham et al., 2017). In this study, both the total PVQ score and subdimension scores were used as independent variables. Participants were classified as having experienced peer victimization if their PVQ score exceeded 2 (Zhu and Lei, 2006). The Cronbach’s *α* for PVQ in this sample was 0.92, indicating high internal consistency.

### Non-suicidal self-injury

NSSI in the past 12 months were assessed using the Chinese version of the Functional Assessment of Self-Mutilation (C-FASM) (Qu et al., 2021). The C-FASM includes: A checklist of 10 NSSI (e.g., cutting, burning, hitting oneself); A list of 15 NSSI functions, evaluating the psychological motivations for NSSI, including: Emotion Regulation, Attention Seeking, Social Avoidance. In this study, NSSI incidence (0 = No, 1 = Yes) and NSSI function scores (emotion regulation, attention seeking, social avoidance) were used as dependent variables. The Cronbach’s *α* for FASM in this sample was 0.91, demonstrating good internal consistency.

### Covariates

To control for potential confounding factors, demographic variables were included as covariates: Age (continuous variable); Ethnicity (Han = 1, Minority = 0); Residence (Urban = 1, Rural = 0); Socioeconomic Status (SES) (parental education level, annual household income).

### Statistical analysis

All data were analyzed using IBM SPSS 26.0, with stratified analyses conducted separately for male and female participants. For descriptive statistics, continuous variables were reported as mean (M) ± standard deviation (SD), while categorical variables were presented as frequencies (*n*) and percentages (%). Chi-square tests (*χ*^2^) were used to compare gender differences in categorical variables, whereas independent samples *t*-tests were employed to examine gender differences in continuous variables. To further investigate the impact of peer victimization (PVQ subdimensions) on NSSI functions (emotion regulation, attention seeking, social avoidance), multiple linear regression was conducted separately for male and female groups. In the regression models: Dependent variable (*Y*): NSSI functions (emotion regulation, attention seeking, and social avoidance); Independent variables (*X*): PVQ scores for physical victimization, social victimization, verbal victimization, and property victimization; Covariates: Age, SES, ethnicity, parental education level. The study performed two regression models: Model I (Baseline Model): This model included only the total peer victimization score to assess its direct predictive effect on NSSI functions. Model II (Adjusted Model): This model extended Model 1 by incorporating age, SES, ethnicity, and other covariates, allowing for the analysis of the independent contribution of peer victimization to NSSI functions. Separate regression analyses were conducted for male and female subgroups, without incorporating interaction terms, to examine gender differences in these relationships. All statistical analyses used two-tailed tests, with significance set at *p* < 0.05. Unstandardized regression coefficients (*β*) were used to interpret the practical impact of independent variables on dependent variables. Cohen’s d was calculated to measure the effect size of gender differences. Additionally, Variance Inflation Factor (VIF) was computed to assess multicollinearity, with VIF <10 considered acceptable. As all questionnaire items were mandatory, there were no missing data, eliminating the need for missing data imputation.

## Results

### Demographic characteristics of participants

This study included a total of 2,343 adolescents with depressive disorders, of whom 517 (22.1%) were male and 1,826 (77.9%) were female. The mean age of males was significantly higher than that of females (*p* < 0.001). Demographic analysis revealed significant gender differences in ethnicity and grade distribution. The proportion of Han ethnicity was significantly higher than that of ethnic minorities (*p* < 0.001). In terms of grade level, the proportion of high school students was significantly higher among males (*p* < 0.001), while females had a higher proportion of middle school students. Other variables, including place of residence, parental education level, and family income, showed no significant gender differences (*p* > 0.05, see Table 1).

**Table 1:** Demographic characteristics of the sample.

Characteristic	Male (*n* = 517)	Female (*n* = 1826)	Total sample (*n* = 2,343)	*p*
Age (year)	15.44 ± ± 1.52	14.87 ± 1.67	14.99 ± 1.65	0.009
Ethnic groups *n* (%)				
Han	492 (95.2)	1,643 (90.0)	2,135 (91.1)	<0.001
Minority	25 (4.8)	183 (10.0)	208 (8.9)	
Residence *n* (%)				
Urban	363 (70.2)	1,217 (66.6)	1,580 (67.4)	0.127
Rural	150 (29.8)	608 (33.4)	763 (32.6)	
Grade *n* (%)				
Primary school	12 (2.3)	79 (4.3)	91 (3.9)	<0.001
Junior high school	229 (44.3)	1,020 (55.9)	1,249 (53.3)	
Senior high school	264 (51.1)	699 (38.3)	963 (41.1)	
College	12 (2.3)	28 (1.5)	40 (1.7)	
Father’s education *n* (%)				
Primary school and below	72 (13.9)	229 (12.5)	301 (12.8)	0.548
Junior high school	190 (36.8)	692 (37.9)	882 (37.6)	
Senior high school	107 (20.7)	420 (23)	527 (22.5)	
Junior college	68 (13.2)	204 (11.2)	272 (11.6)	
Bachelor’s degree or above	80 (15.5)	281 (15.4)	361 (15.4)	
Mother’s education *n* (%)				
Primary school and below	125 (24.2)	407 (22.3)	532 (22.7)	0.298
Junior high school	162 (31.3)	647 (35.4)	809 (34.5)	
Senior high school	112 (21.7)	341 (18.7)	453 (19.3)	
Junior college	58 (11.2)	225 (12.3)	283 (12.1)	
Bachelor’s degree or above	60 (11.6)	206 (11.3)	266 (11.4)	
Annual household income *n* (%)				
Less than ¥50,000	125 (24.2)	489 (26.8)	614 (26.2)	0.218
¥50,000–¥100,000	91 (17.6)	317 (17.4)	408 (17.4)	
¥100,000–¥200,000	92 (17.8)	252 (13.8)	344 (14.7)	
More than ¥200,000	48 (9.3)	170 (9.3)	218 (9.3)	
Uncertain	161 (31.1)	598 (32.7)	759 (32.4)	

### Preliminary analysis of peer victimization and NSSI differences by gender

Peer victimization was assessed using the PVQ scale. The overall victimization rate was significantly higher in females (78.09%) than in males (73.69%) (*p* = 0.036). When examining specific dimensions of victimization, females had significantly higher rates and scores for social victimization compared to males (*p* < 0.001). However, no significant gender differences were observed in the prevalence or scores of physical, verbal, or property victimization (*p* > 0.05, see Tables 2, 3).

**Table 2:** Comparison of peer victimization and NSSI incidence.

Outcome		Gender		Total	*χ2*	*p*
		Male (*n*/%)	Female (*n*/%)			
Physical victimization	No	322 (62.28)	1,102 (60.35)	1,424 (60.78)	0.631	0.427
	Yes	195 (37.72)	724 (39.65)	919 (39.22)		
Social victimization	No	275 (53.19)	772 (42.28)	1,047 (44.69)	19.414	0.000**
	Yes	242 (46.81)	1,054 (57.72)	1,296 (55.31)		
Verbal victimization	No	177 (34.24)	665 (36.42)	842 (35.94)	0.834	0.361
	Yes	340 (65.76)	1,161 (63.58)	1,501 (64.06)		
Property victimization	No	291 (56.29)	1,008 (55.20)	1,299 (55.44)	0.192	0.662
	Yes	226 (43.71)	818 (44.80)	1,044 (44.56)		
Victimized (Yes/No)	No	136 (26.31)	400 (21.91)	536 (22.88)	4.421	0.036*
	Yes	381 (73.69)	1,426 (78.09)	1807 (77.12)		
NSSI (Yes/No)	No	199 (38.49)	362 (19.82)	561 (23.94)	77.094	0.000**
	Yes	318 (61.51)	1,464 (80.18)	1782 (76.06)		

*p < 0.05, **p < 0.01.

**Table 3:** Comparison of PVQ and FASM scores.

Outcome		Male	Female	*t*	*p*	95%CI
PVQ	Physical victimization	3.21 ± 4.74	2.99 ± 4.23	0.93	0.35	−0.24 to 0.67
	Social victimization	4.40 ± 5. 35	5.82 ± 5.90	−5.23	0.000**	−1.96 to −0.89
	Verbal victimization	6.00 ± 5.39	6.32 ± 5.76	−1.18	0.24	−0.86 to 0.21
	Property victimization	3.69 ± 4.79	3.77 ± 4.80	−0.31	0.76	−0.54 to 0.40
	Total score	17.30 ± 17.07	18.91 ± 16.85	−1.91	0.06	−3.29 to 0.04
FASM	Emotion regulation function	9.85 ± 4.93	12.33 ± 5.04	−9.91	0.000**	−2.97 to −1.99
	Attention seeking.	9.49 ± 4.40	10.22 ± 4.30	−3.39	0.001**	−1.15 to −0.31
	Social avoidance	6.09 ± 3.02	6.58 ± 3.06	−3.32	0.001**	−0.79 to −0.20

*p < 0.05, **p < 0.01.

NSSI behavior and its functions were assessed using the FASM scale. The prevalence of NSSI was significantly higher in females (80.18%) than in males (61.51%) (*p* < 0.001). In terms of NSSI functionality scores, females scored significantly higher than males in emotion regulation (*p* < 0.001), attention-seeking (*p* = 0.001), and social avoidance (*p* = 0.001), indicating that females were more likely to engage in NSSI for these purposes (see Table 3).

### Regression analyses: gender-specific predictive effects of peer victimization on NSSI functions

Multiple linear regression was conducted to examine the impact of different types of peer victimization on NSSI as an emotion regulation strategy, with separate analyses for males and females.

In males, social victimization (*β* = 1.213, *p* = 0.036) and verbal victimization (*β* = 1.764, *p* = 0.031) significantly positively predicted the use of NSSI for emotion regulation. This suggests that males who experience these forms of victimization are more likely to engage in NSSI to regulate emotions. Other victimization types (overall victimization, physical, and property victimization) showed no significant effects.

In females, physical victimization (*β* = 0.768, *p* = 0.005) and social victimization (*β* = 0.873, *p* < 0.001) significantly predicted NSSI for emotion regulation. Verbal victimization was significant in Model I (*p* < 0.001) but became non-significant after controlling for confounding variables in Model II (*p* = 0.182), suggesting that its effect may be partially mediated by other factors such as residence or age. Overall victimization showed a reversed significance in Model II (*p* = 0.005), possibly due to confounding factors. These findings indicate that females are more influenced by physical and social victimization when using NSSI for emotion regulation, whereas males are more sensitive to social and verbal victimization (see Table 4).

**Table 4:** Gender differences in the impact of peer victimization on the emotional regulation function of NSSI.

Gender	Outcome	Model I				Model II				
		*β*	*t*	*p*	95%CI	*β*	*t*	*p*	95%CI	VIF
Male	Constant	8.230	22.457	0.000**	7.511 ~ 8.948	13.705	5.484	0.000**	8.807 ~ 18.603	–
	Victimized (Yes/No)	−1.078	−1.184	0.237	−2.863 ~ 0.707	−0.941	−1.027	0.305	−2.735 ~ 0.854	3.697
	Physical victimization	0.760	1.355	0.176	−0.339 ~ 1.859	0.57	1.008	0.314	−0.539 ~ 1.679	1.710
	Social victimization	1.058	1.867	0.063	−0.053 ~ 2.169	1.213	2.099	0.036*	0.080 ~ 2.346	1.892
	Verbal victimization	1.279	2.353	0.019*	0.213 ~ 2.344	1.764	2.167	0.031*	0.168 ~ 3.359	3.393
	Property victimization	−0.003	−0.006	0.995	−1.037 ~ 1.031	−0.006	−0.012	0.991	−1.065 ~ 1.053	1.634
Female	Constant	10.503	51.35	0.000**	10.103 ~ 10.904	13.094	10.526	0.000**	10.656 ~ 15.532	–
	Victimized (Yes/No)	−0.063	−0.142	0.887	−0.939 ~ 0.812	−0.031	−0.07	0.005**	−0.907 ~ 0.845	2.616
	Physical victimization	0.816	2.993	0.003**	0.282 ~ 1.351	0.768	2.804	0.005**	0.231 ~ 1.305	1.376
	Social victimization	0.882	3.123	0.002**	0.328 ~ 1.435	0.873	2.841	0.000**	0.271 ~ 1.475	1.763
	Verbal victimization	1.298	4.59	0.000**	0.744 ~ 1.852	1.238	3.636	0.182	0.570 ~ 1.905	2.055
	Property victimization	0.373	1.383	0.167	−0.156 ~ 0.901	0.366	1.335	0.944	−0.172 ~ 0.905	1.428

*p < 0.05, **p < 0.01.

Regarding the impact of peer victimization on NSSI for attention-seeking, regression results showed: In males, physical victimization (*β* = 1.182, *p* = 0.017), social victimization (*β* = 1.24, *p* = 0.015), and verbal victimization (*β* = 1.436, *p* = 0.044) were all significant predictors of NSSI for attention-seeking. Neither overall victimization nor property victimization showed significant associations. In females, physical victimization (*β* = 0.705, *p* = 0.003), social victimization (*β* = 0.738, *p* = 0.005), and verbal victimization (*β* = 1.195, *p* < 0.001) significantly predicted NSSI for attention-seeking. Property victimization was marginally significant (*p* = 0.091), suggesting a potential influence. Both genders were significantly affected by physical, social, and verbal victimization, but males exhibited generally higher coefficients, indicating a stronger motivation for using NSSI as an attention-seeking strategy in response to these victimization experiences (see Table 5).

**Table 5:** Gender differences in the impact of peer victimization on the attention-seeking function of NSSI.

Gender	Outcome	Model I				Model II				
		*β*	*t*	*p*	95%CI	*β*	*t*	*p*	95%CI	VIF
Male	Constant	7.804	24.325	0.000**	7.175 ~ 8.432	11.69	5.343	0.000**	7.402 ~ 15.978	–
	Victimized (Yes/No)	−1.113	−1.396	0.163	−2.675 ~ 0.450	−0.914	−1.14	0.255	−2.485 ~ 0.657	3.697
	Physical victimization	1.376	2.803	0.005**	0.414 ~ 2.338	1.182	2.387	0.017*	0.211 ~ 2.153	1.710
	Social victimization	1.069	2.154	0.032*	0.096 ~ 2.041	1.24	2.45	0.015*	0.248 ~ 2.232	1.892
	Verbal victimization	0.986	2.073	0.039*	0.054 ~ 1.919	1.436	2.015	0.044*	0.039 ~ 2.833	3.393
	Property victimization	0.037	0.079	0.937	−0.869 ~ 0.942	0.017	0.036	0.972	−0.910 ~ 0.944	1.634
Female	Constant	8.844	50.41	0.000**	8.500 ~ 9.188	8.555	8.025	0.000**	6.466 ~ 10.645	–
	Victimized (Yes/No)	−0.539	−1.407	0.160	−1.289 ~ 0.212	−0.543	−1.418	0.156	−1.294 ~ 0.208	2.616
	Physical victimization	0.682	2.917	0.004**	0.224 ~ 1.141	0.705	3.002	0.003**	0.245 ~ 1.165	1.376
	Social victimization	0.592	2.446	0.015*	0.118 ~ 1.067	0.738	2.804	0.005**	0.222 ~ 1.254	1.763
	Verbal victimization	0.977	4.027	0.000**	0.501 ~ 1.452	1.195	4.097	0.000**	0.624 ~ 1.767	2.055
	Property victimization	0.311	1.343	0.18	−0.143 ~ 0.764	0.398	1.689	0.091	−0.064 ~ 0.859	1.428

*p < 0.05, **p < 0.01.

For the role of peer victimization in NSSI as a social avoidance strategy, the results indicated gender-specific differences: In males, physical victimization (*β* = 0.972, *p* = 0.005) and social victimization (*β* = 0.885, *p* = 0.012) significantly predicted NSSI for social avoidance. Verbal victimization was not significant in Model II (*p* = 0.105). In females, physical victimization (*β* = 0.528, *p* = 0.002), social victimization (*β* = 0.632, *p* = 0.001), verbal victimization (*β* = 0.551, *p* = 0.008), and property victimization (*β* = 0.564, *p* = 0.001) all significantly predicted NSSI for social avoidance. These findings suggest that males’ social avoidance function of NSSI is primarily influenced by physical and social victimization, whereas females are influenced by a broader range of victimization types, including physical, social, verbal, and property victimization (see Table 6).

**Table 6:** Gender differences in the impact of peer victimization on the social avoidance function of NSSI.

Gender	Outcome	Model I				Model II				
		*β*	*t*	*p*	95%CI	*β*	*t*	*p*	95%CI	VIF
Male	Constant	5.168	23.259	0.000**	4.732 ~ 5.603	6.669	4.379	0.000**	3.684 ~ 9.654	–
	Victimized (Yes/No)	−0.913	−1.656	0.098	−1.994 ~ 0.168	−0.879	−1.575	0.116	−1.972 ~ 0.215	3.697
	Physical victimization	1.020	2.999	0.003**	0.353 ~ 1.686	0.972	2.819	0.005**	0.296 ~ 1.647	1.710
	Social victimization	0.760	2.21	0.028*	0.086 ~ 1.433	0.885	2.514	0.012*	0.195 ~ 1.576	1.892
	Verbal victimization	0.269	0.816	0.415	−0.377 ~ 0.915	0.805	1.623	0.105	−0.167 ~ 1.778	3.393
	Property victimization	0.019	0.059	0.953	−0.608 ~ 0.646	0.089	0.272	0.786	−0.556 ~ 0.735	1.634
Female	Constant	5.499**	44.367	0.000**	5.256 ~ 5.742	6.604	8.767	0.000**	5.128 ~ 8.081	-
	Victimized (Yes/No)	−0.288	−1.063	0.288	−0.818 ~ 0.243	−0.274	−1.011	0.312	−0.804 ~ 0.257	2.616
	Physical victimization	0.539**	3.263	0.001**	0.215 ~ 0.863	0.528	3.179	0.002**	0.202 ~ 0.853	1.376
	Social victimization	0.567**	3.315	0.001**	0.232 ~ 0.903	0.632	3.398	0.001**	0.268 ~ 0.997	1.763
	Verbal victimization	0.478**	2.789	0.005**	0.142 ~ 0.814	0.551	2.674	0.008**	0.147 ~ 0.955	2.055
	Property victimization	0.535**	3.276	0.001**	0.215 ~ 0.856	0.564	3.391	0.001**	0.238 ~ 0.890	1.428

*p < 0.05, **p < 0.01.

## Discussion

This study explores the relationship between peer victimization and non-suicidal self-injury (NSSI) in adolescents with depressive disorders, with a particular focus on gender differences. The results show that the prevalence of NSSI is significantly higher in females than in males. Moreover, the impact of different forms of peer victimization on NSSI varies by gender: social victimization and verbal victimization are more strongly associated with NSSI in males, while physical victimization and social victimization have a greater impact on NSSI in females. The following section provides a detailed discussion of the effects of different forms of peer victimization on NSSI.

### Emotion regulation function

The study found that emotion regulation is the most commonly endorsed function of NSSI, with females scoring significantly higher than males in this regard. This result is consistent with previous research, which suggests that adolescent females are more likely than males to use NSSI as a mechanism for coping with negative emotions, possibly due to greater emotional sensitivity and a stronger tendency to internalize stress (Wang et al., 2025).

In males, social victimization and verbal victimization significantly predicted emotion regulation-related NSSI. Research suggests that social victimization may undermine self-esteem and hinder help-seeking behaviors, while verbal victimization may reinforce negative self-perceptions, leading to maladaptive coping strategies such as NSSI (van Geel et al., 2015; Vergara et al., 2019; Baldwin et al., 2019). Specifically, the relationship between social victimization and self-esteem damage has been validated in multiple studies. Adolescents who experience various forms of victimization (such as physical, relational, and cyberbullying) often exhibit higher levels of anxiety and depression, which are closely related to a decline in self-esteem (Lai et al., 2023; Becker et al., 2017). Social victimization may lead adolescents to form negative self-evaluations, which further exacerbates their psychological distress (Taylor et al., 2013). In males, social victimization is particularly likely to damage self-esteem and make it more difficult for them to seek help during emotional distress. These psychological impacts may prompt them to adopt maladaptive coping strategies, such as NSSI (Sinclair et al., 2012).

Verbal victimization also significantly affects negative self-perceptions in male adolescents. Studies have found that verbal victimization is closely associated with negative and depressive cognition in adolescents, particularly in males (Sinclair et al., 2012). Furthermore, verbal victimization may increase the risk of NSSI by affecting their emotion regulation abilities. The reinforcement of negative self-perceptions may lead male adolescents to be more likely to use NSSI as an emotion regulation strategy (Chen et al., 2025).

In females, physical victimization and social victimization are the primary predictors of emotion regulation-related NSSI. Rather than increasing pain tolerance, repeated exposure to physical victimization may lead to emotional numbing or emotional desensitization, thus lowering the threshold for engaging in NSSI (Hooley et al., 2010). Social victimization, particularly exclusion and isolation, may exacerbate feelings of loneliness and social anxiety, prompting females to use NSSI to regulate negative emotions (Lee et al., 2024). Therefore, in clinical practice, interventions for female adolescents exposed to physical and social victimization should focus on enhancing emotion regulation skills. For male adolescents, interventions can focus on alleviating the psychological impact of social and verbal victimization and promoting the development of adaptive coping strategies.

### Attention-seeking function

Both males and females demonstrated significant associations between physical, social, and verbal victimization and attention-seeking NSSI, although the regression coefficients were generally higher for males. In males, physical victimization may lead them to express distress through overt behaviors, particularly when emotional expression is socially constrained (Keohane and Richardson, 2018; Vickery, 2021). Social and verbal victimization may further drive the use of NSSI as a signal for support, consistent with the interpersonal theory of NSSI, which posits that self-injury can function as a means of eliciting care from others (Klonsky, 2007).

In females, although physical, social, and verbal victimization also predicted attention-seeking NSSI, the strength of these associations was smaller. This may reflect gender differences in communication patterns (Sampasa-Kanyinga and Hamilton, 2015). Females may be more inclined to express their need for help verbally rather than through overt self-injury, unless the victimization is prolonged (Lloyd-Richardson et al., 2007). Nevertheless, social victimization remains an important predictor of attention-seeking NSSI in females, which may be related to social exclusion and interpersonal distress.

In clinical intervention, attention-seeking NSSI should focus on improving assertive communication skills, especially for male adolescents experiencing multiple forms of peer victimization, and strengthening the development of social support networks.

### Social avoidance function

Social avoidance-driven NSSI shows significant gender differences in the association with types of victimization. In males, only physical and social victimization significantly predicted this function. Physical victimization may increase the tendency to withdraw to avoid further harm, while social victimization may promote avoidance by reducing distress from hostile peer interactions (Macalli et al., 2021). In females, all four forms of victimization (physical, social, verbal, and property victimization) are important predictors of social avoidance-related NSSI. Although property victimization does not involve direct physical harm, it may have symbolic relational meaning in collectivist cultural contexts, representing exclusion or humiliation. Particularly when possessions hold social value, property victimization may indirectly trigger NSSI, especially when it co-occurs with relational aggression (Li and Jiang, 2018; Zhang et al., 2020).

Social victimization significantly affects both genders, which can be explained by the need-to-belong theory (Miao et al., 2020): social exclusion threatens the universal human motivation for social connection, leading to emotional distress and maladaptive coping behaviors such as NSSI. Although gender norms may influence how distress is expressed (for example, males may be more likely to suppress emotional expression, while females may exhibit greater interpersonal sensitivity), the fundamental psychological impact of social exclusion may be consistent across genders. This universality may explain why social victimization is an important predictor for both male and female adolescents in our study.

From a prevention perspective, addressing social avoidance-driven NSSI may require both individual-level interventions (such as social skills training) and systemic interventions (such as school-based anti-bullying programs), with particular attention to females experiencing multiple forms of victimization.

## Limitations

Despite its valuable contributions, this study has several limitations. This study used a cross-sectional design, preventing causal inferences. Future research should employ longitudinal designs or real-time monitoring techniques such as ecological momentary assessment to verify whether peer victimization directly leads to an increase in NSSI behaviors. The study primarily relied on self-reported data, which may be subject to social desirability bias. Future research could incorporate multiple assessment methods, such as clinical interviews or behavioral assessments, to enhance data accuracy. Fourth, the gender distribution in our sample was unbalanced, with females significantly outnumbering males (77.9% vs. 22.1%). This disparity may have influenced the stability of gender-specific regression estimates and limits the generalizability of gender comparison results. Fifth, the present study did not assess the severity of depressive symptoms at the time of data collection. Symptom severity could influence both perceived peer victimization and the likelihood of engaging in NSSI, potentially confounding the associations observed. Future studies should include standardized severity measures to control for this effect. The sample consisted primarily of adolescents with depressive disorders, which may limit generalizability to other populations. Future studies should expand the sample to examine the universality of the peer victimization-NSSI relationship across different populations.

## Conclusion

Overall, this study demonstrates that peer victimization is a significant predictor of NSSI, with notable gender differences in its impact. Male adolescents are more influenced by social and verbal victimization, whereas female adolescents are more affected by physical and social victimization. These findings are further supported by real-time assessment data, reinforcing the importance of gender-sensitive approaches in understanding NSSI mechanisms. Effective NSSI interventions should account for gender-specific risk factors and recognize how different forms of victimization impact mental health to develop more precise prevention and intervention strategies.

## Data Availability

The original contributions presented in the study are included in the article/supplementary material, further inquiries can be directed to the corresponding authors.
